# TRAF3IP2 as a novel inflammatory biomarker for coronary artery disease: development and validation of a multimodal prediction model

**DOI:** 10.3389/fendo.2025.1644403

**Published:** 2025-10-20

**Authors:** Peibing Ge, Tianyi Ni, Mengjie Liu, Qiuyao Du, Changjiang Xu, Tingting Hu, Yang Gu, Hailang Liu, Jin Geng

**Affiliations:** Department of Cardiology, The Affiliated Huaian No.1 People’s Hospital of Nanjing Medical University, Huaian, Jiangsu, China

**Keywords:** coronary artery disease, atherosclerosis, TRAF3IP2, biomarker, nomogram model, inflammation

## Abstract

**Background:**

Coronary artery disease (CAD) is currently among the leading cardiovascular diseases with considerable morbidity/mortality worldwide. While inflammation drives atherosclerosis, clinically actionable biomarkers remain elusive. The role of TRAF3IP2, a proinflammatory adaptor molecule, in the pathogenesis and prediction of coronary artery disease warrants systematic investigation. The purpose of this study was to explore the role of TRAF3IP2 in coronary artery disease and to develop and validate a nomogram for predicting the risk of coronary artery disease.

**Methods:**

GSE12288 gene expression profiles were downloaded from the Gene Expression Omnibus database, and key genes and pathways involved in CAD (n=222) were identified. LASSO and multivariate logistic regression analyses were applied to investigate the risk factors for severe coronary artery stenosis in a clinical cohort (n=280). A nomogram model was developed to predict CAD, and the clinical utility of the nomogram model was evaluated using calibration curves and decision curve analysis (DCA).

**Results:**

Multiple bioinformatics tools revealed that TRAF3IP2 expression was higher in patients with CAD than in controls. Moreover, TRAF3IP2 is involved in the cellular response to inflammation, which is a basic process of atherosclerosis. Clinical data from a total of 280 patients were retrospectively reviewed for our study. Sex (OR 0.446 [0.230–0.863], p=0.017), diabetes history (OR 2.099 [1.131–3.896], p=0.019), phosphoremia (OR 0.252 [0.065–0.972], p=0.045) and TRAF3IP2 (OR 1.040 [1.004–1.076], p=0.027) were independent risk factors for atherosclerosis. The nomogram was composed of these factors, and the calibration curves and DCA curve showed that the model has great potential for clinical utility.

**Conclusions:**

In summary, this study demonstrated that TRAF3IP2 could be a potential biomarker for CAD. A nomogram composed of sex, diabetes history, phosphoremia, and TRAF3IP2 expression may predict the risk of CAD.

## Introduction

Atherosclerotic cardiovascular disease is now the leading cause of death globally (1). Atherosclerosis is the basic pathological process of atherosclerotic cardiovascular diseases. Atherosclerosis is characterized by inflammation of the arterial walls (2) and ultimately decreases the inflow of distal organs, resulting in tissue or cell atrophy/necrosis. This process begins early in life (3). Atherosclerotic plaques form in the intima. In the early stage of atherosclerosis, low-density lipoprotein (LDL) particles accumulate in the intima. Afterward, monocytes enter the intima. After entering the intima, monocytes can mature into macrophages. Thereafter, macrophages bind lipoprotein particles and form foam cells. Smooth muscle cells can also migrate into the intima. Atherosclerotic plaques ultimately develop (4). However, the exact mechanism underlying this process remains to be elucidated.

Multiple factors are believed to be involved in the development of atherosclerosis, including hereditary and environmental factors (5). TRAF3IP2 (TRAF3 Interacting Protein 2) is a proinflammatory adapter molecule. Evidence has shown that TRAF3IP2 plays an important role in the process of atherosclerosis (6). TRAF3IP2 is a vital regulator of angiogenesis (7). Targeting TRAF3IP2 significantly decreases the levels of IL6, IL8, and IL1β (7). TRAF3IP2 gene deletion downregulates the expression of extracellular matrix, matrix metalloproteinase (MMP2), IL-6 and IL-18 and attenuates cardiac hypertrophy and fibrosis (8). An animal study demonstrated that the aortic plaque area and plaque necrotic area were significantly decreased in TRAF3IP2-knockout mice (9).

Using multiple network databases, we conducted a molecular characteristic analysis to assess the potential function of TRAF3IP2 in the diagnosis and prognosis of atherosclerosis. Differentially expressed genes (DEGs), functional enrichment and protein–protein interaction network analyses were performed to determine the function of TRAF3IP2 in atherosclerosis. To further confirm the role of TRAF3IP2 in coronary heart disease, least absolute shrinkage and selection operator (LASSO) regression and multivariate logistic regression were used to restrict the selection of potential markers of atherosclerosis in patients with coronary heart disease. The findings of this study indicate that TRAF3IP2 can function as a biomarker of atherosclerosis.

## Materials and methods

### Access to public data

The GEO database (http://www.ncbi.nlm.nih.gov/geo) is a public database that contains many high-throughput resources, including gene expression data, microarray data, and chromatin immunoprecipitation (ChIP)-Seq data (10). We used the ‘GEO query’ package of R software (version 4.3.2) to download the GSE12288 microarray data from the GEO database (https://www.ncbi.nlm.nih.gov/geo/query/acc.cgi?acc=GSE12288). The GSE12288 dataset is based on the GPL96 platform and contains 222 samples, including 110 patients with CAD and 112 partially matched controls without CAD (11). To eliminate technical bias while preserving inherent biological variability between groups, we first filtered out the genes with expression values<10 in at least 25% of the samples. After log2 transformation was performed, the median and interquartile range were adjusted. Moreover, we removed probes lacking corresponding annotation information and averaged the expression levels of duplicate genes.

### Differentially expressed genes identification

The raw GSE12288 data were read by R software (version 4.3.2). DEGs were screened by the “limma” package. The cutoff points for statistically significant DEGs were defined as |log2FC|> 1 and a P value< 0.05. Scatter plots and Violin plots were created to compare TRAF3IP2 expression in peripheral blood samples from patients with CAD and controls without CAD. Moreover, the associations between TRAF3IP2 expression and different CADi (Duke CAD index, CADi, a validated angiographical measure of the extent of coronary atherosclerosis that correlates with outcome) were identified with Spearman’s correlation.

### Gene annotation

GeneCodis (https://genecodis.genyo.es/), a web tool that can integrate results from different omics methods to perform advanced gene set analysis of small RNAs, TFs and methylation sites, was introduced in our study (12). We used an online platform to perform functional annotation and an analysis of TRAF3IP2.

### Construction and analysis of the PPI network and GO and KEGG analyses

The TRAF3IP2-interacting proteins were identified by intersecting the interacting proteins retrieved from GeneMANIA (http://www.genemania.org/), BioGRID (http://thebiogrid.org/), and the STRING database (https://string-db.org/). Common genes were subsequently subjected to Gene Ontology (GO) and Kyoto Encyclopedia of Genes (KEGG) pathway enrichment analyses with R software.

### Study population

**Figure 1:**
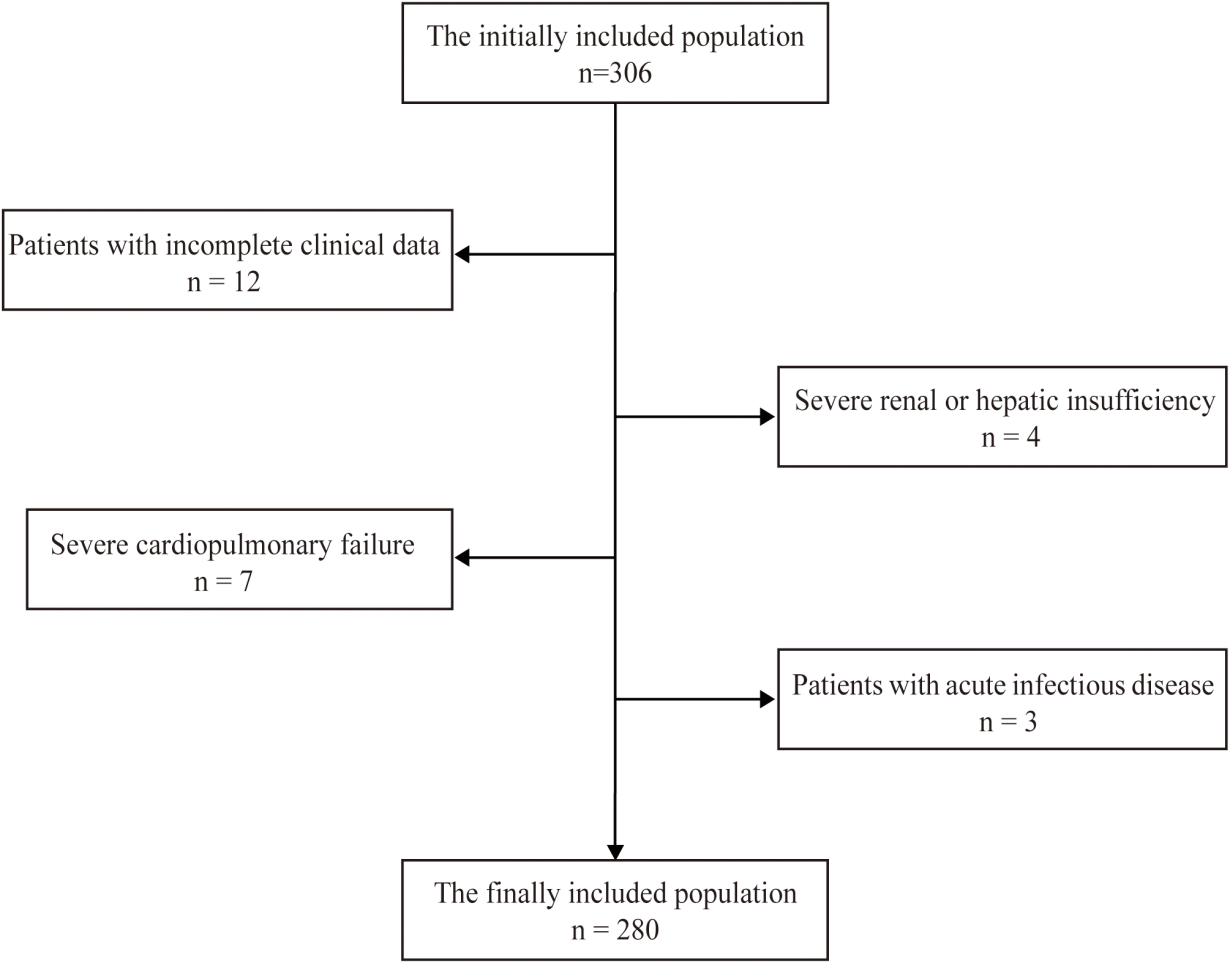
The flowchart for screening the research population.

The participants were recruited from inpatients admitted to The Affiliated Huaian No. 1 People’s Hospital of Nanjing Medical University from September 2023 to December 2023. The inclusion criteria were as follows: the patient was between 18 and 80 years old, underwent coronary angiography in the Cardiology Department, and provided consent. The exclusion criteria included (1) patients lacking relatively complete clinical data; (2) patients with severe hepatic/renal insufficiency; (3) patients with severe cardiopulmonary failure; and (4) patients with acute infection. A total of 280 patients were included in our study. All methods were conducted in accordance with relevant guidelines and regulations. All the experimental protocols were approved by the Ethics Committee of our hospital. Informed consent was obtained from all the subjects and/or their legal guardian(s). The flowchart for screening the research population is shown in **Figure 1**.

### Study design

Two experienced experts performed coronary angiography on the patients. According to their coronary angiography results, the patients included in our study were divided into two groups: the mild stenosis group and the severe stenosis group. The mild stenosis group included patients with mild stenosis (only one vessel with less than 70% occlusion on coronary angiography by visual estimation). The severe stenosis group included those with severe stenosis (at least one vessel with more than 70% occlusion on coronary angiography by visual estimation).

### Baseline characteristics

These participants underwent a careful clinical evaluation at baseline. The following characteristics were assessed: history of smoking, alcohol use, hypertension, and diabetes, and several health metrics (body mass index (BMI), blood pressure, creatinine level, fasting blood glucose level, glycosylated hemoglobin level, total cholesterol level, triglyceride level, low-density lipoprotein–cholesterol level, and high-density lipoprotein–cholesterol level). All physiological and biochemical indicators were measured from venous blood samples collected after a 12-hour fast.

### Measurement of TRAF3IP2

TRAF3IP2 expression levels were determined using an enzyme-linked immunosorbent assay (ELISA). After the blood samples were taken, they were maintained at 4°C for 24 hours and then centrifuged at 12,000 rpm for 10 minutes at 4°C. The supernatant was aliquoted (1–2 mL) and stored at -80°C until further analysis. Serum TRAF3IP2 levels were measured using a TRAF3IP2 ELISA kit (RX100044H) purchased from Ruixin Biotech (Quanzhou, China). In accordance with the product manual, 50 μL of standard solution with varying concentrations was added to the standard wells, 50 μL of sample diluent was added to the zero wells, and 50 μL of the sample to be tested was added to the sample wells. Blank wells were marked on the plate. After 100 μL of HRP-conjugated detection antibody was added, the mixtures were incubated for 60 min at 37°C in a water bath. A substrate mixture was then added for color development, and then the mixture was incubated at 37°C for 15 min after five washing cycles. Finally, 50 μL of termination solution was added to terminate the reaction. The optical density at 450 nm (OD 450) was read using a microplate reader. Each sample was set up in three replicate wells.

### Statistical analysis

The statistical analysis was performed using SPSS Statistics 27.0. The normality of the variable distribution was assessed, and the Levene test was used to verify the homogeneity of variance. Continuous variables with a normal distribution are displayed as the mean ± SD. Independent sample t tests were used for comparisons between the two groups. One-way ANOVA was used for comparisons between multiple groups. Nonnormally distributed continuous variables are displayed as medians and IQRs (interquartile ranges) [M (Q1, Q3)] and were compared with the Mann–Whitney U test and the Wilcoxon rank-sum test. Categorical variables are presented as counts and frequencies and were compared with the chi-square test and Fisher’s exact test. Spearman’s correlation test was applied to assess the relationships between TRAF3IP2 and iCAD. LASSO and multivariate logistic regression analyses were performed to identify risk factors for severe coronary artery stenosis. Based on these results, we developed a nomogram model incorporating TRAF3IP2 and traditional factors to predict the risk of CAD in patients with severe stenosis using R 4.3.1 software. Calibration curves were constructed to assess the accuracy and consistency of the model. Decision curve analysis (DCA) was used to evaluate clinical utility. Receiver operating characteristic (ROC) analysis is an accurate method for assessing diagnostic tests (13). All tests were 2-sided, and a P value<0.05 was considered to indicate statistical significance.

## Results

### The expression characteristics of TRAF3IP2 in CAD in GSE12288

**Figure 2:**
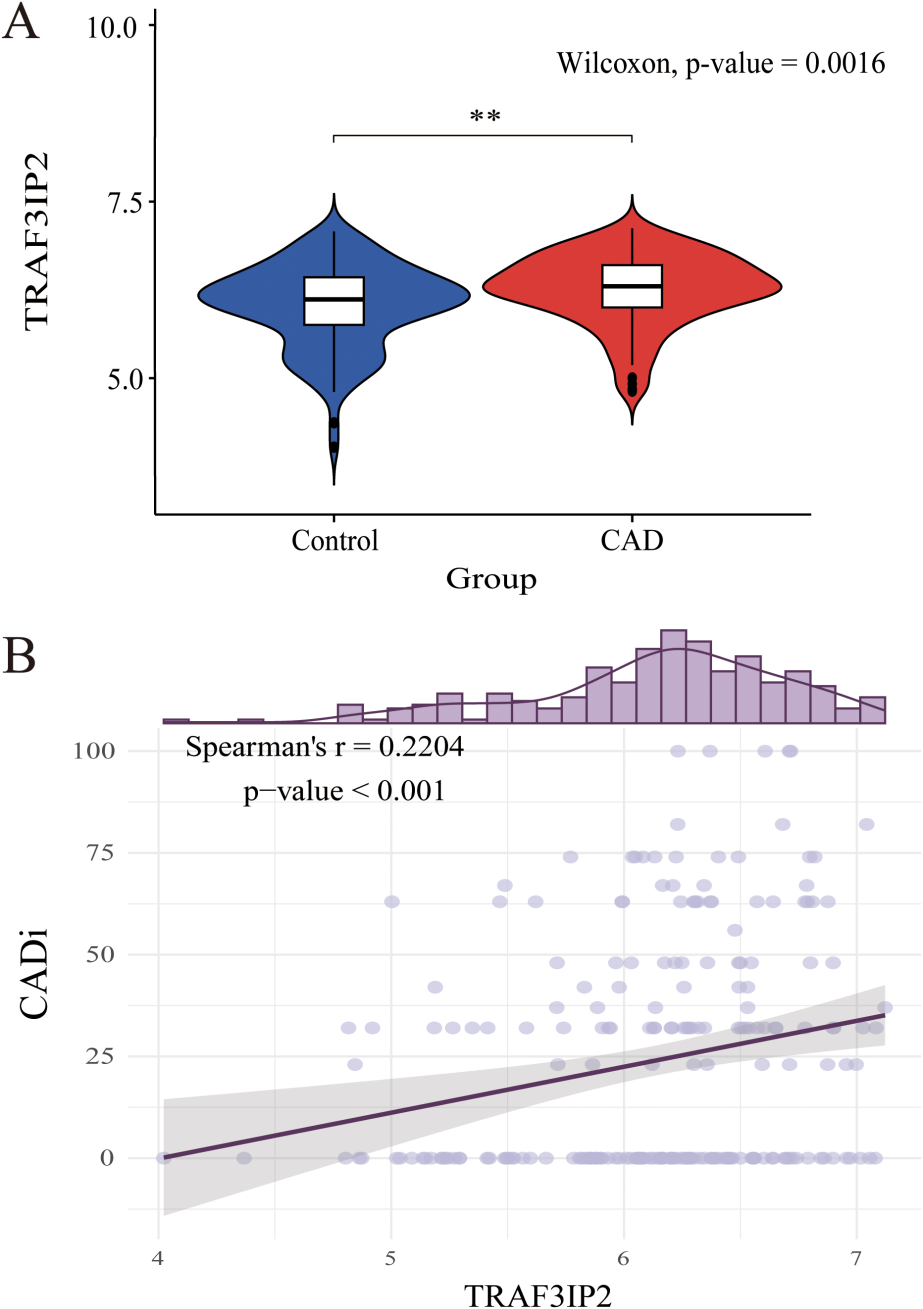
**(A)** Differential expression analysis of TRAF3IP2 between the CAD disease group and the control group in GSE12288. **(B)** Spearman’s correlation between TRAF3IP2 and CADi. **indicates statistical significance at p < 0.05.

GSE12288 was downloaded and analyzed. Compared with that in controls without CAD, TRAF3IP2 expression was higher in patients with CAD. The Violin plot is shown in **Figure 2A**. Moreover, Spearman’s correlation analysis revealed that the TRAF3IP2 expression level was positively correlated with the CADi (Spearman’s r=0.22; p<0.001) (**Figure 2B**). These results indicate that TRAF3IP2 plays a role in the development of CAD.

### Gene annotation of TRAF3IP2

**Figure 3:**
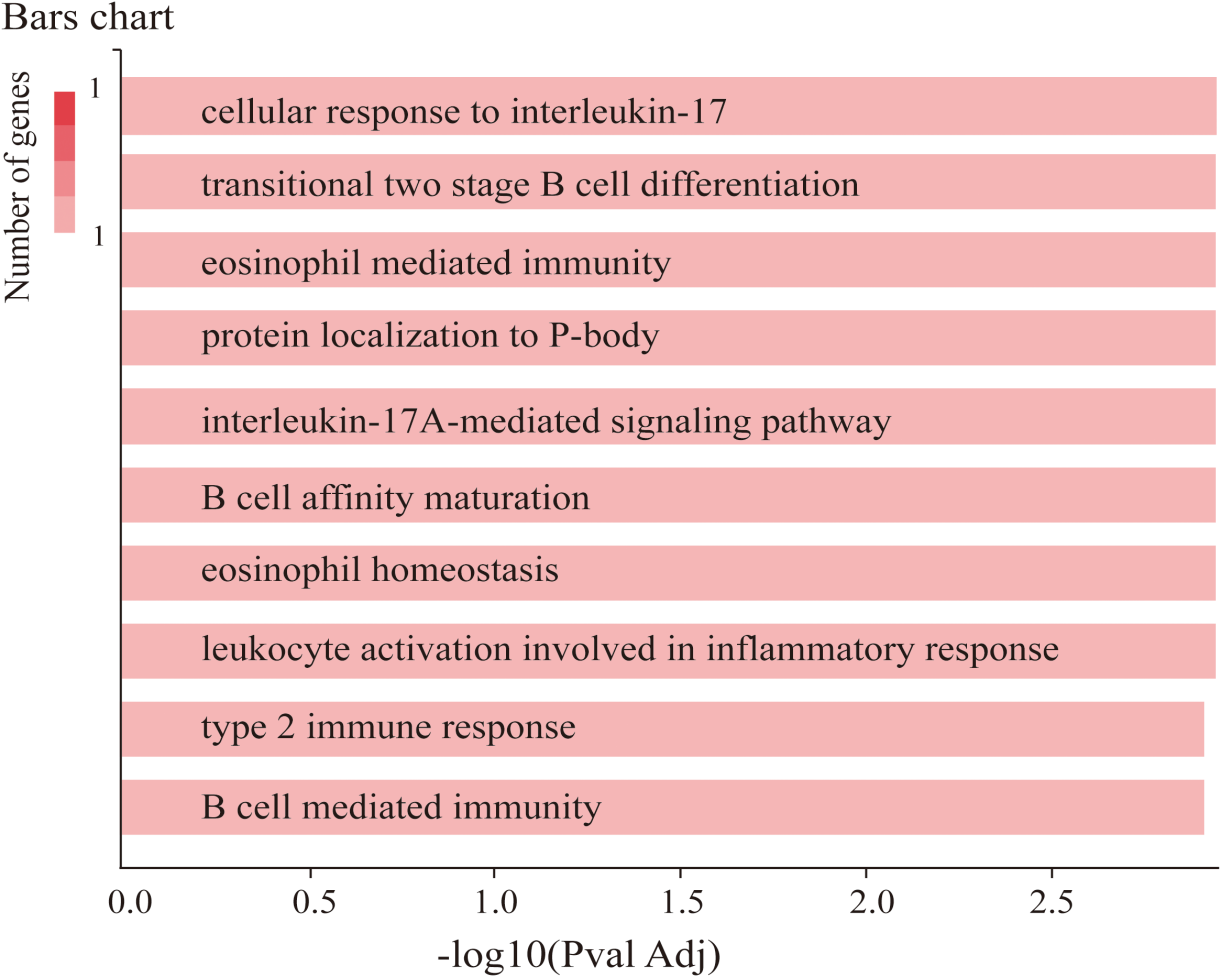
Co-annotation analysis of TRAF3IP2 with respect to functional and regulatory elements using GeneCodis 4.

To verify the function of TRAF3IP2 in CAD, GeneCodis, a web tool, was used in our study. The results revealed that TRAF3IP2 is involved in the cellular response to interleukin-17, the interleukin-17A-mediated signaling pathway, transitional two-stage B-cell differentiation, eosinophil-mediated immunity, protein localization to the P-body, B-cell affinity maturation and so forth (**Figure 3**).

### Construction and analysis of the PPI network and GO and KEGG analyses

**Figure 4:**
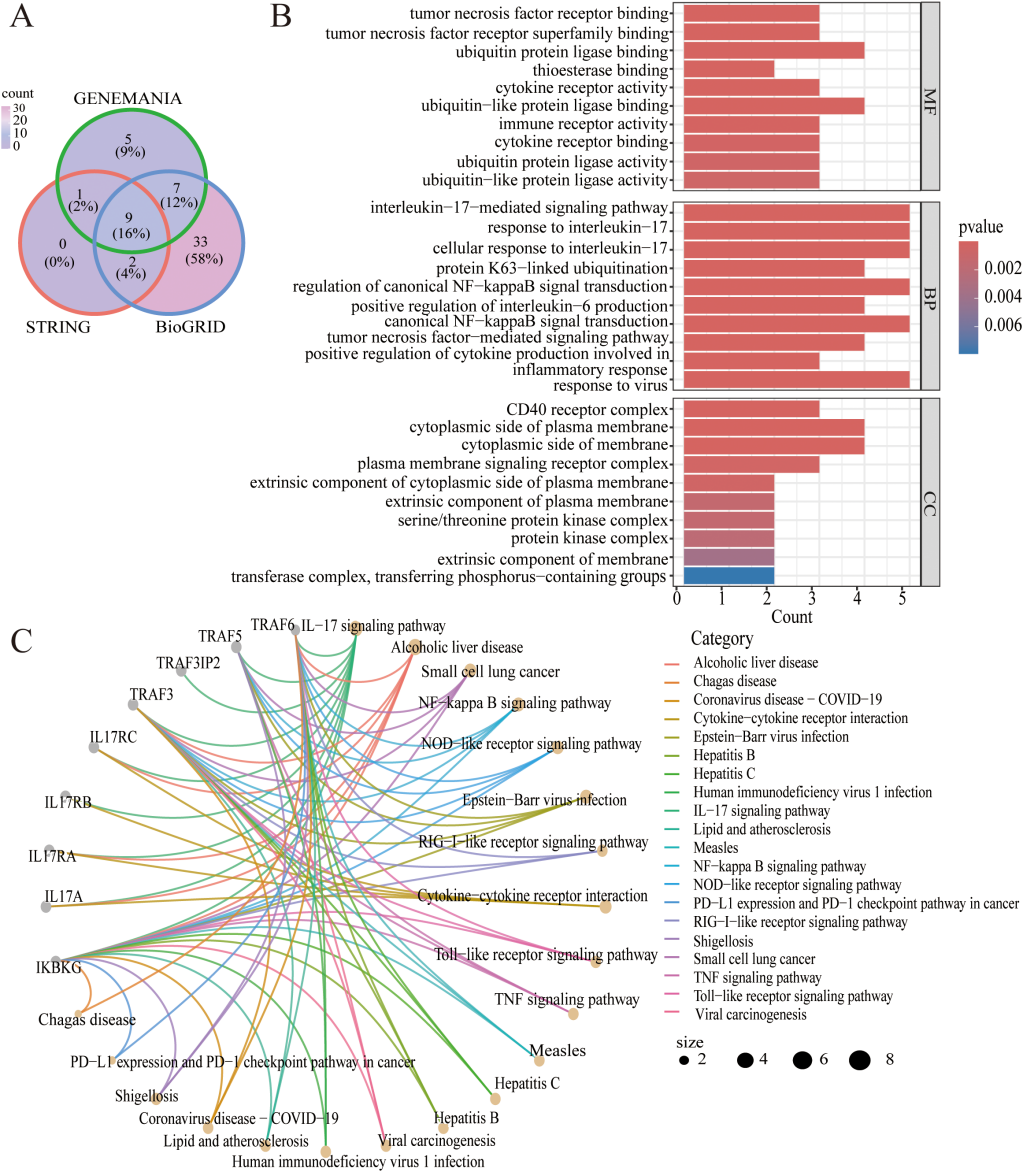
**(A)** Venn diagram of the intersection of TRAF3IP2-interacting proteins in the GeneMANIA, BioGRID and STRING platforms. **(B)** GO analysis of the intersection of proteins that interact with TRAF3IP2. **(C)** KEGG analysis of the intersection of proteins that interact with TRAF3IP2.

Nine common genes (IKBKG IL17A IL17RA IL17RB IL17RC TRAF3 TRAF3IP2 TRAF5 TRAF6) were discovered on GeneMANIA, BioGRID and STRING (**Figure 4A**). As shown in **Figure 4B**, the results of the GO analysis were enriched mainly in the interleukin-17 signaling pathway, alcoholic liver disease, the NF-kappa B signaling pathway, and the NOD-like receptor signaling pathway. KEGG analyses revealed pathways enriched in the interleukin-17-mediated signaling pathway, the CD40 receptor complex, and tumor necrosis factor receptor binding (**Figure 4C**).

### Baseline characteristics of the patients

The mean age was 64.24 ± 11.98 years (age range: 55 to 73 years), and 69.64% were men. Compared with the mild stenosis group, the severe stenosis group ​​had a higher proportion of​​ male patients, smokers, and individuals with low blood phosphorus levels or high TRAF3IP2 expression. There was no difference in the number of patients with a history of drinking, hypertension, or diabetes, and no difference in BMI, LDL-C or FBG. The baseline characteristics of the patients included in our study are summarized in **Table 1**.

**Table 1:** Comparison of baseline data between the mild stenosis group and the severe stenosis group.

Baseline data	All patients (n=280)	Mild stenosis group (n=112)	Severe stenosis group (n=168)	t/Z/ $X^{2}$	P
Age (years)	64.24 ± 11.98	66.50 (57.25,73.00)	65.50 (55.00,72.00)	-0.885	0.376
male[n (%)]	195 (69.64)	62 (54.39)	133 (79.17)	18.019	<0.001***
smoker [n (%)]	91 (32.5)	24 (21.05)	67 (39.88)	10.43	0.001**
drinking[n (%)]	57 (20.36)	18 (15.79)	39 (23.21)	2.115	0.146
hypertension [n (%)]	181 (64.64)	73 (64.04)	108 (64.29)	0.023	0.878
diabetes [n (%)]	80 (28.57)	25 (21.93)	55 (32.74)	3.573	0.059
BMI	24.87 ± 2.58	24.40 (23.60,26.37)	24.34 (23.66, 25.95)	-0.445	0.656
Cr (μmol/L)	74.86 ± 30.62	67.45 (55.85,80.13)	71.05 (61.28,85.23)	-1.963	0.050
P (mmol/L)	1.20 ± 0.21	1.24 ± 0.18	1.17 ± 0.23	2.555	0.011**
TC (mmol/L)	3.80 ± 1.05	3.65 (3.05,4.40)	3.64 (2.94,4.50)	-0.288	0.774
TG (mmol/L)	1.72 ± 1.22	1.32 (1.02,1.88)	1.48 (1.03,2.12)	-1.447	0.148
LDL-C (mol/L)	2.27 ± 0.94	2.10 (1.58,2.93)	2.16 (1.47,2.93)	-0.004	0.997
FBG (mmol/L)	5.97 ± 2.23	5.14 (4.59,6.01)	5.26 (4.79,6.65)	-1.686	0.092
HbA1c/%	6.70 ± 1.26	6.25 (5.80,7.10)	6.30 (5.80,7.50)	-0.379	0.704
TRAF3IP2	30.38 ± 7.68	28.98 ± 7.68	31.33 ± 7.56	-2.559	0.011**

*** is P<0.001, ** is P<0.05.

### Screening and verification of diagnostic markers

**Figure 5:**
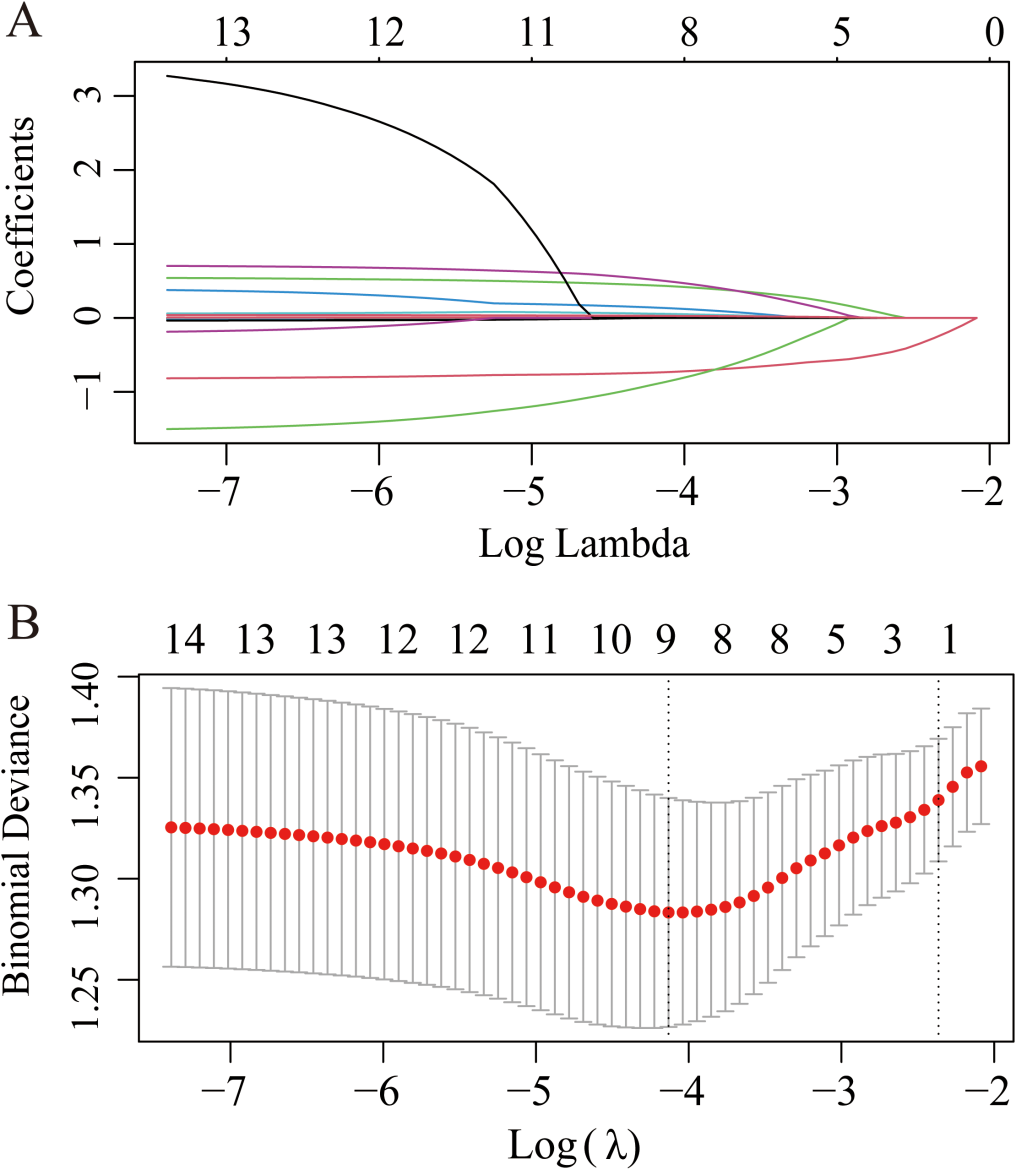
LASSO regression plot of screening for the most significant risk factors for severe coronary artery occlusion. **(A)** The LASSO coefficient profile plot. **(B)** The cross-validation error curve.

A least absolute shrinkage and selection operator (LASSO) regression model was used to determine the most significant risk factors for severe coronary artery occlusion. λ is taken as the minimum value. Tenfold cross-validation via minimum criteria was used to select the tuning parameter (lambda) in the LASSO model. A coefficient profile plot was created against the log(lambda) sequence. The screening procedure is shown in **Figure 5A, B**. To avoid overfitting of the model, the partial regression coefficient of the independent variable is gradually reduced to 0. In this study, the λ _min value (0.09401152) of the vertical dashed line on the right side of the LASSO regression curve was chosen as the optimal model. By using the least absolute shrinkage and selection operator (LASSO) regression model, we identified 9 risk factors, namely, age, sex, smoking history, diabetes history, serum creatinine level, phosphoremia, total cholesterol (TC) level, triglyceride (TG) level, and TRAF3IP2 expression. Multivariate logistic regression analysis including the aforementioned nine risk factors was conducted to predict the presence of atherosclerosis. The results revealed that sex, diabetes history, phosphoremia and TRAF3IP2 expression (OR 1.040 [1.004–1.076], p=0.027) were independent risk factors for atherosclerosis (**Table 2**).

**Table 2:** Multivariate logistic regression of coronary stenosis severity.

Clinical characteristic	B	Standard error	*OR* (95% *CI*)	P
Age	-0.006	0.013	0.994 (0.969,1.019)	0.618
Sex (Male)	0.808	0.337	2.244 (1.159,4.345)	0.017
Smoker	0.529	0.318	1.697 (0.911,1.163)	0.096
Diabetes	0.742	0.315	2.099 (1.131,3.896)	0.019
Serum creatinine	0.005	0.006	1.005 (0.994,1.017)	0.365
Blood phosphorus	-1.377	0.688	0.252 (0.065,0.972)	0.045
Total cholesterol (TC)	0.228	0.141	1.256 (0.952,1.658)	0.107
Triglyceride (TG)	0.091	0.147	1.096 (0.821,1.462)	0.535
TRAF3IP2	0.039	0.018	1.040 (1.004,1.076)	0.027

**Figure 6:**
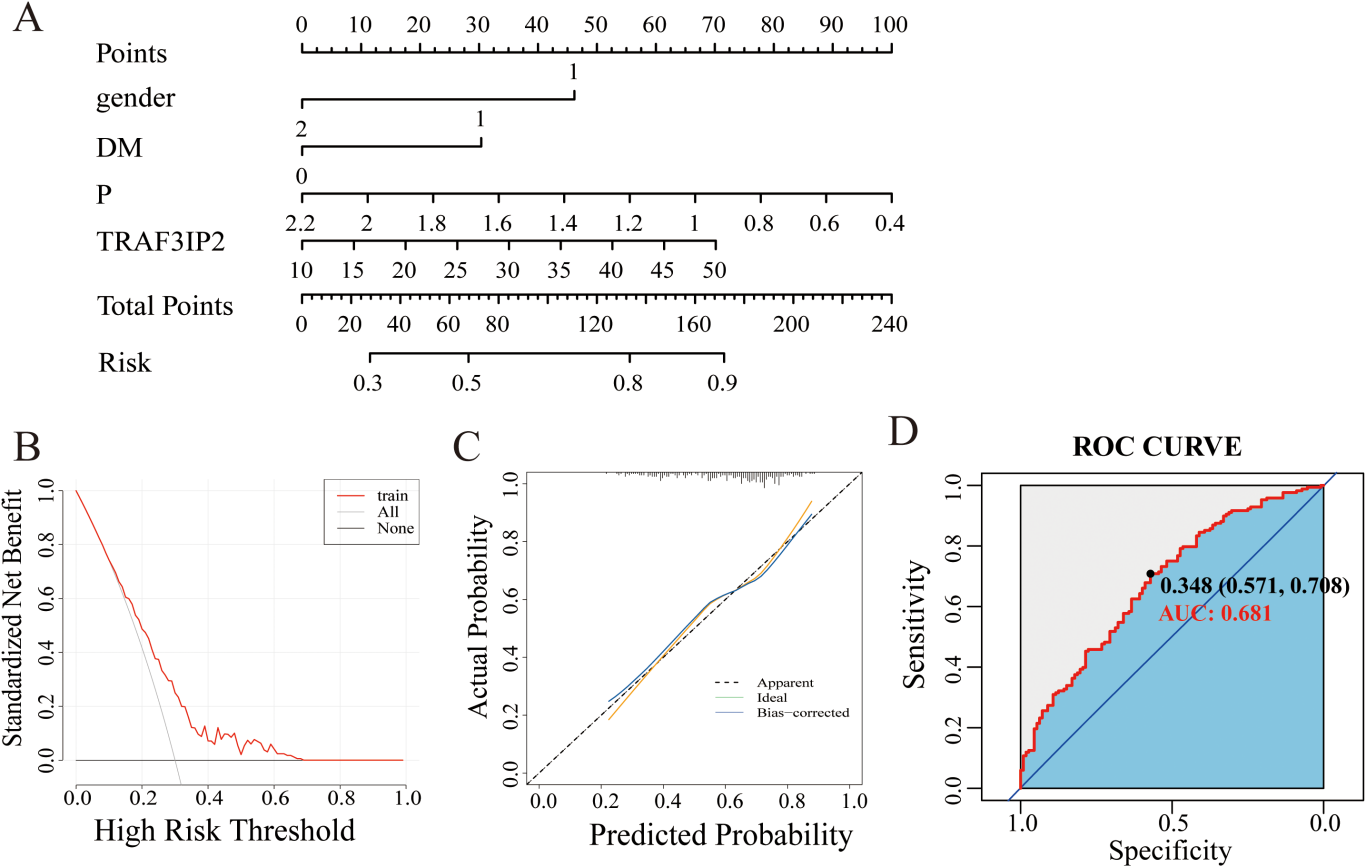
**(A)** Nomogram model of the main factors influencing the prediction of CAD with severe stenosis. **(B)** Calibration curve of the nomogram model. **(C)** DCA curve of the nomogram model. **(D)** The ROC curve of the nomogram model.

A nomogram based on constructed LASSO regression and multivariate logistic regression models was constructed to predict the probability of CAD in patients with severe stenosis (**Figure 6A**). The results revealed higher risk scores (70) for patients with TRAF3IP2 expression (≥50). Visualization of risk factors can predict the individual risk of CAD. First, each unique risk factor was projected upward to the first row of the scale to obtain a score for each element; then, the scores for the four risk factors were summed to obtain a total score. The higher the total score is, the higher the risk of CAD for the individual.

The calibration curve showed that the probability predicted by the model was well-matched with the actual probability, and the Hosmer-Lemeshow test yielded an *X* ^2^ value of 4.9752 and a p value of 0.7602, indicating a good fit for the model (**Figure 6B**). The DCA curve showed that the model has great potential for clinical utility (**Figure 6C**).

The area under the curve for discriminative power was 0.681 (95% CI: 0.618–0.745). The cutoff value was 34.80, with a sensitivity of 70.8% and a specificity of 57.1% (**Figure 5D**).

## Discussion

In this study, we used patients with mild stenosis as the control group and developed a multivariable diagnostic model incorporating TRAF3IP2 expression to predict severe coronary artery stenosis. The goal is to identify a clinically noninvasive alternative for evaluating the degree of coronary artery stenosis. Bioinformatics analysis revealed that TRAF3IP2 is involved in the cellular response to inflammation. LASSO and multivariate logistic regression analyses revealed that sex, diabetes history, phosphoremia status and TRAF3IP2 expression were independent risk factors for atherosclerosis. Nomograms are widely used to construct diagnostic prediction models and survival models for many common diseases, including diabetes and cancer (14, 15). A CAD risk prediction model was constructed with the nomogram model. The calibration curves and the DCA curve showed that the model is reliable. Our findings extend the current knowledge by identifying TRAF3IP2 as a master regulator bridging IL-17-mediated inflammation with clinical CAD manifestations.

Cardiovascular diseases and stroke are life-threatening diseases that affect individuals worldwide. Atherosclerosis, which is a chronic inflammatory disease of the arterial wall, is the basic process of numerous cardiovascular diseases and stroke. The CANTOS trial demonstrated that targeting the interleukin-1β innate immune pathway and administering anti-inflammatory therapy led to a significantly lower rate of recurrent cardiovascular events (16). The COLCOT trial suggested that reducing inflammation with 0.5 mg of colchicine once daily may reduce the risk of cardiovascular disease (17). The LoDoCo trial revealed that among patients with a recent myocardial infarction, low-dose daily colchicine led to a significantly lower risk of cardiovascular events (18). Despite statins effectively decreasing LDL-C levels and circulating inflammation markers (19), a residual inflammatory risk may still persist, which contributes to the occurrence of MACEs among individuals receiving lipid-lowering treatment (20). Indeed, the PROVE-IT (21), IMPROVE-IT (22), and SPIRE-1/2 (23) trials revealed that approximately 29%, 33%, and 47% of participants were at risk of residual inflammation. Non-HDL-C is acknowledged as the main contributor to a residual risk of ASCVD in patients on statin therapy with controlled LDL-C levels (24). Ridker et al. (25) reported that among patients receiving contemporary statins, inflammation assessed by high-sensitivity CRP was a stronger predictor of future cardiovascular events and death than cholesterol assessed by LDL-c was.

Age, sex, smoking history, diabetes history, and TC and TG levels were conventional risk factors for atherosclerosis. Notably, the results of this study indicated that higher levels of TRAF3IP2 and lower phosphoremia level were important risk factors for the development of atherosclerosis. In both the GSE12288 database and the patients included in our study, the expression of TRAF3IP2 was higher in the patients with CAD than in the controls. A positive correlation has been identified between TRAF3IP2 levels and CADi, suggesting that TRAF3IP2 may play a role in the development of atherosclerosis.

Atherosclerosis is a chronic inflammatory disease primarily driven by excessive inflammatory immune responses (26). Previous studies have shown that TRAF3IP2 is a critical component of the inflammatory pathway. It is related mainly to the interleukin-17 signaling pathway, the NF-κB signaling pathway, and the NOD-like receptor signaling pathway and is an upstream regulator of IKK/NF-κB and JNK/AP-1, activating multiple downstream oxidative stress-responsive proinflammatory pathways, thereby playing a critical role in inflammation and tissue injury (27). Moreover, TRAF3IP2 acts as a cytoplasmic adaptor molecule and can induce the expression of multiple proinflammatory mediators, such as TNFα, CXCL1, VCAM1, ICAM1, TIMP1, RECK, and ADAM17, which play pivotal roles in the development and vulnerability of atherosclerotic plaques in ApoE(-/-) mice (9). Silencing TRAF3IP2 in GBM cells can lead to a reduction in the expression of proinflammatory and proangiogenic cytokines, including IL-1β, IL-6, and IL-8. Ridker et al. (16). further indicated that TRAF3IP2 directly interacts with IL-17 receptors and CD40, activating NF-κB through TRAF6-dependent ubiquitination. The positive correlation between serum TRAF3IP2 and IL-6 levels in our cohort study further supports its upstream regulatory role: TRAF3IP2→ NF-κB →IL-6 amplification cascade. Silencing TRAF3IP2 in GBM cells can lead to a reduction in the expression of proinflammatory and proangiogenic cytokines, including IL-1β, IL-6, and IL-8.

Liao (28) revealed that endothelial dysfunction was the initiator of atherogenesis. Balachandar Venkatesan et al. (29) reported that TRAF3IP2 mediates high glucose-induced endothelial dysfunction. The results indicated that knockdown of TRAF3IP2 inhibited high glucose-induced IKKβ and JNK phosphorylation; inhibited p65 and c-Jun nuclear translocation; and inhibited the expression of NF-κB-dependent proinflammatory cytokines, chemokines, and adhesion molecules, thereby contributing to the alleviation of endothelial dysfunction and a reduction in the risk of atherosclerosis. Additionally, knockdown of TRAF3IP2 reversed oxLDL-induced impaired vasodilatation and markedly attenuated oxLDL-induced coronary artery endothelial cell death ex vivo (30).

The proliferation and migration of human aortic vascular smooth muscle cells (HAVSMCs) constitute another key factor leading to atherosclerosis (31). TRAF3IP2 is a critical signaling intermediate in IL-17A signaling. IL-17A promotes the migration and proliferation of HAVSMCs in a TRAF3IP2-dependent manner First, TRAF3IP2 activates its downstream targets, including NF-κB, AP-1, and p38MAPK. It subsequently induces NLRP3 expression in smooth muscle cells, ultimately driving their proliferation and migration (32). Additionally, Anthony et al. (33) reported that TRAF3IP2 can also mediate IL-18-induced cardiac fibroblast migration and differentiation.

Furthermore, the risk of CAD is likely to be higher in specific populations than in the general population. For example, the study by Giosiana et al. revealed increased LCB and an impaired innate immune profile in FH patients with subclinical atherosclerosis, and both were independently associated with atherosclerotic injury (34). In addition, certain autoimmune diseases, such as psoriasis, rheumatoid arthritis, and systemic lupus erythematosus, may increase the risk of CVD, which may be due to chronic inflammation (35). A population-based study revealed that the incidence rate of cardiovascular disease among patients with autoimmune disease is approximately 1.5 times higher per 1,000 patient-years than that in patients without autoimmune disease (36). Currently, the findings of several studies have suggested that variants of specific genes may increase susceptibility to CAD (37). Based on this perspective, the relevance of TRAF3IP2 as a biomarker not only in general CAD populations but also in genetically predisposed individuals suggests that TRAF3IP2 can be explored as a prognostic or stratification marker, even in high-risk populations.

To our knowledge, our study is the first to reveal that TRAF3IP2 is related to atherosclerosis in humans. Our study revealed elevated serum TRAF3IP2 levels as an independent predictor of CAD severity, which is consistent with its established role as an adapter protein in proinflammatory signaling (9, 30).

In the present study, a nomogram model was constructed and revealed satisfactory discrimination and calibration ability. Furthermore, the model can successfully stratify patients into different risk groups. Patients with higher risks may need more aggressive clinical evaluations more frequently.

The results of our study should be interpreted with respect to several limitations. First, this was a retrospective study conducted at a single center, which might generate bias. Second, other atherosclerosis risk factors, such as exercise, family history of dyslipidemia, history of cerebral infarction or cerebral hemorrhage, history of previous myocardial infarction or angina pectoris, history of stent implantation, and pulse wave velocity (a commonly used surrogate marker for endothelial dysfunction and early atherosclerosis), were not considered. Additionally, the dynamic variations in these risk factors, such as smoking, alcohol consumption, sleep state and dietary habits, were not included in the risk factor analysis. Finally, the mechanism by which TRAF3IP2 mediates CAD remains poorly understood, as it is based solely on bioinformatics analyses and previous findings. Hence, causal relationships need to be explored and validated through multidimensional cellular and animal experiments. Therefore, in subsequent studies, researchers should expand the sample size and incorporate more clinical information, as well as additional noninvasive biomarkers. Moreover, cell or animal experiments involving gene overexpression or knockout will be conducted to validate the relevant pathways and molecular mechanisms involved.

## Conclusions

In this study, we screened and identified four CAD-associated independent risk factors, including sex, diabetes history, phosphoremia, and TRAF3IP2. Moreover, we visualized these four risk factors using a nomogram, which can be used as a clinical tool for personalized screening. Future studies are warranted to validate our nomogram in different populations.

## Data Availability

The original contributions presented in the study are included in the article/supplementary material. Further inquiries can be directed to the corresponding author.
